# Change in Neurocognitive Function in Patients Who Receive CAR-T Cell Therapies: A Steep Hill to Climb

**DOI:** 10.3390/ph17050591

**Published:** 2024-05-06

**Authors:** Evlampia Strongyli, Paschalis Evangelidis, Ioanna Sakellari, Maria Gavriilaki, Eleni Gavriilaki

**Affiliations:** 1 Hematology Department and Bone Marrow Transplant (BMT) Unit, G. Papanicolaou Hospital, 57010 Thessaloniki, Greece; evlabia1@gmail.com (E.S.); ioannamarilena@gmail.com (I.S.); 2 Second Propedeutic Department of Internal Medicine, Hippocration Hospital, Aristotle University of Thessaloniki, 54642 Thessaloniki, Greece; pascevan@auth.gr; 3 1st Department of Neurology, AHEPA University Hospital, Aristotle University of Thessaloniki, 54636 Thessaloniki, Greece; mariagavri6@yahoo.gr

**Keywords:** apraxia, cognition, CAR-T, ICANS, lymphoma, memory, myeloma, neurotoxicity

## Abstract

Immunotherapy with chimeric antigen receptor T (CAR-T) cell therapies has brought substantial improvement in clinical outcomes in patients with relapsed/refractory B cell neoplasms. However, complications such as cytokine release syndrome (CRS) and immune effector cell-associated neurotoxicity syndrome (ICANS) limit the therapeutic efficacy of this treatment approach. ICANS can have a broad range of clinical manifestations, while various scoring systems have been developed for its grading. Cognitive decline is prevalent in CAR-T therapy recipients including impaired attention, difficulty in item naming, and writing, agraphia, and executive dysfunction. In this review, we aim to present the diagnostic methods and tests that have been used for the recognition of cognitive impairment in these patients. Moreover, up-to-date data about the duration of cognitive impairment symptoms after the infusion are presented. More research on the risk factors, pathogenesis, preventive measures, and therapy of neurocognitive impairment is crucial for better outcomes for our patients.

## 1. Introduction

Chimeric antigen receptor T (CAR-T) cell therapies have revolutionized the management of patients with relapsed/refractory B lymphoproliferative malignancies, producing remarkable clinical responses [1]. To date, four CAR-T products are available for the treatment of lymphomas and B-acute lymphoblastic leukemia (B-ALL): lisocabtagene maraleucel for diffuse large B cell lymphoma (DLBCL), primary mediastinal large B cell lymphoma (PMBCL), and grade 3B follicular lymphoma (FL); axicabtagene ciloleucel for DLBCL, PMBCL, and FL; brexucabtagene autoleucel for relapsed or refractory mantle cell lymphoma (MCL) and B-ALL; and tisagenlecleucel for DLBCL and B-ALL in young adult patients [2]. Recently, two products were approved for administration in patients with relapsed/refractory multiple myeloma (MM): ciltacabtagene autoleucel and idecabtagene vicleucel [3,4]. However, CAR-T therapeutics brought novel complications and toxicities following their infusion [5]. These include cytopenias, diffuse intravascular coagulation, infections, and endothelial injury syndromes, such as cytokine release syndrome (CRS) and immune effector cell-associated neurotoxicity syndrome (ICANS) [6,7,8,9]. American Society for Transplantation and Cellular Therapy (ASTCT) criteria are used in everyday clinical practice for CRS severity grading [10]. Management of CRS includes administration of tocilizumab (interleukin-6 receptor blocker), steroids, and empiric antimicrobial therapy, due to the difficult differential diagnosis from infections.

ICANS onset is usually acute and transitory, while its clinical manifestations are diverse. It may present as dizziness, headaches, delirium, seizures, impairment of motor skills, hallucinations, asterixis, and tremors [11]. Moreover, cognitive deficits can be another presentation of ICANS. Expressive or global aphasia, dysgraphia, disorientation, reduced attention, amnesia, and apraxia are prevalent in these patients [12]. The immune effector cell-associated encephalopathy (ICE) score, constituted from the following five points: (1) orientation to date, city, and hospital; (2) naming of three items; (3) ability to follow simple commands; (4) counting backward from 100 by 10; and (5) writing of a standard phrase, is used in everyday clinical practice for ICANS severity evaluation. However, the ICE score is not sensitive to the detection of cognitive deficits [13]. Another scoring system, Common Terminology Criteria for Adverse Events (CTCAE), has been used in some cases for neurotoxicity grading, but it was not specially designed for patients who receive CAR-T cell infusions [14].

This review aims to summarize the existing literature providing evidence on the prevalence, diagnosis, clinical manifestations, and pathophysiology of neurocognitive impairment post-CAR-T cell infusions. Moreover, diagnostic tests and methods for the detection of neurocognitive deficits in CAR-T cell therapy patients are thoroughly presented. In addition to these, we summarize the pathophysiology of ICANS, in which neurocognitive impairment can be attributed. A PubMed search was performed to identify relevant articles published in English, using the following medical terms: “cognition”, “neurocognitive”, “memory”, “ICANS”, and “CAR-T”. Early recognition of cognitive deficits is considered crucial for better outcomes in these patients in order to improve their quality of life.

## 2. ICANS: Grading, Immunopathology, and Treatment Approach

ICANS incidence has been described as high as 67% in patients who receive CAR-T cell immunotherapy for acute lymphoblastic leukemia, and up to 62% in those with lymphomas, constituting a common CAR-T cell-associated toxicity [15]. However, the reported incidence of neurotoxicity post-axicabtagene ciloleucel infusion has been reported as high as 87% [16]. The median time of ICANS onset is 4 days post CAR-T infusion, while most cases of neurotoxicity have been described within 3 weeks after the infusion of CAR-T products [17,18]. According to the ASTCT grading system, ICANS manifestations can be divided into four grades [10]. Grade 1 (ICE score: 7–9) patients exhibit mild disorientation, inattentiveness, and mild expressive and/or receptive dysarthria (patients can communicate). Grade 2 (ICE score: 3–6) patients are characterized by moderately impaired consciousness levels but are responsive to voice. Grade 3/4 (grade 3, ICE score: 0–2; grade 4, ICE score: 0) patients (severe ICANS) have significant dysarthria, respond only to noxious or tactile stimuli, and might exhibit seizures. Severe ICANS might lead to intracerebral hemorrhage and cerebral edema, which in some cases might be fatal.

A disturbance in the permeability and integrity of the blood–brain barrier (BBB), as shown by high levels of CAR-T cells, and other T cells (CD4+, CD8+), is observed in patients with ICANS [19]. Activation of macrophage/monocyte cells by CAR-T cell products results in the release of multiple cytokines and other inflammatory molecules, such as angiopoietin-2, granulocyte-macrophage colony-stimulating factor (GM-CSF), interferon-gamma (IFN-γ), interleukin-1β (IL-1β), interleukin-6 (IL-6), interleukin-10 (IL-10), and interleukin-15 (IL-15) [20,21]. These cytokines lead to neuroinflammation and breakdown of the BBB, each in a unique way. GM-CSF plays a pivotal role in the manifestations of ICANS [22]. The knockout of genes implicated in the transcription of GM-CSF diminishes the inflammation mediated by cytokine release following CAR-T cell product infusion [23]. GM-CSF results in an increase in the levels of TNF-α, which increases the expression of leukocyte adhesion molecules, such as intercellular adhesion molecule 1 (ICAM-1) and vascular cell adhesion molecule 1 (VCAM-1) in the endothelial cells of the BBB, participating in the migration of monocytes and other inflammatory cells in the central nervous system [24]. Lenzilumab, a recombinant monoclonal antibody against GM-CSF, with potential immunomodulating activity, has been investigated as a prophylactic agent for CRS and ICANS in a phase 1/2 trial [25]. Increased serum and cerebrospinal fluid (CSF) levels of IFN-γ have been reported in CAR-T recipients who developed ICANS in comparison to those who did not [26]. IFN-γ induces the expression of ICAM-1 and VCAM-1 by endothelial cells, participating in neuroinflammation [27].

**Figure 1:**
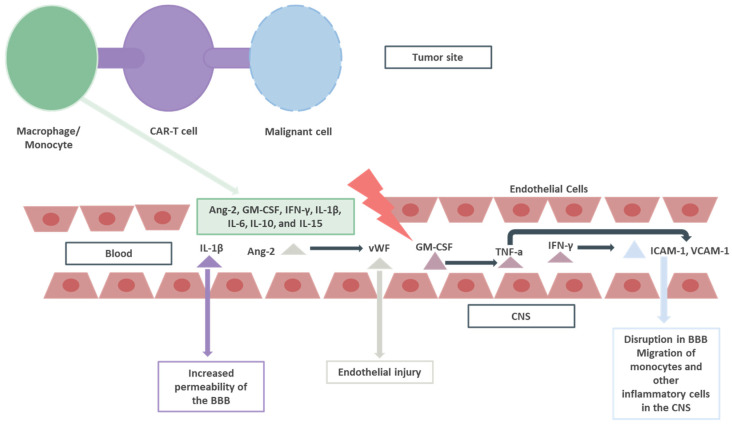
Basic insights of ICANS immunopathology. ICANS: immune effector cell-associated neurotoxicity syndrome; CAR-T: chimeric antigen receptor T; Ang-2: Angiopoietin-II; GM-CSF: granulocyte-macrophage colony-stimulating factor; IFN-γ: interferon-gamma; ΙL-1β: interleukin-1β; IL-6: interleukin-6; IL-10: interleukin-10; IL-15: interleukin-15; ICAM-1: intercellular adhesion molecule 1; VCAM-1: vascular cell adhesion molecule 1; CNS: central nervous system; BBB: blood brain barrier.

IL-1β also increases the permeability of the BBB, reducing the expression of tight junction proteins by astrocytes [28]. IL-6 mediates inflammatory response both during CRS and ICANS [29]. Tocilizumab has been shown to be effective in the management of CRS, but data also support that this agent failed to protect mice from ICANS development [30,31]. Endothelial injury is implicated in the development of ICANS. Angiopoietin-II and von Willebrand factor (vWF) have been found to be increased in those with severe ICANS (grade 4) [32]. Furthermore, an increased angiopoietin-2:angiopoietin-1 ratio has been reported in patients with severe ICANS [32]. Endothelial activation, stress index score (EASIX), and the modified EASIX (m-EASIX), as markers of endothelial injury, have been found as predictors of severe ICANS [33,34]. Other markers such as increased serum levels of IL-2 and IL-5 in the early period post-CAR-T cell infusion (day 3) were found to be predictors of severe ICANS development [35]. In summary, systemic inflammation results in endothelial cells’ activation and disruption in the BBB, resulting in increased cytokine levels in the CSF (Figure 1).

Close monitoring of patients with neurotoxicity is crucial [36]. Neurological examination, including calculation of the ICE score and assessment of motor function, should be performed twice a day. Supportive care and administration of corticosteroids constitute the standard of care in patients with ICANS [36]. For the treatment of grade > 2 ICANS, dexamethasone or methylprednisolone are suggested. Interestingly, tocilizumab might worsen ICANS symptoms, as has been shown in Cohort-3 of the Zuma-1 trial [37]. Moreover, the treatment approach to ICANS is different in patients with concurrent CRS. For severe neurotoxicity cases, admission of the patient to the intensive care unit (ICU) and close cooperation with critical care physicians are important.

## 3. Cognitive Outcomes following CAR-T Cell Therapy

Various approaches have been used in clinical practice for the measurement and evaluation of cognition in patients who receive CAR-T cell therapies. Neurological examination after the onset of cognitive symptoms and frequent assessment until resolution have been reported [38]. ICANS grading, ΙCE recording, and monitoring of neurological symptoms have been found to be crucial, especially in the first two weeks post-infusion [39,40,41,42,43,44,45,46]. In the first two weeks following CAR-T cell therapy cognitive impairment is considered a manifestation of ICANS, which in some cases might be fatal, as described in various case reports [13,40,42,46]. Neurocognitive deficits include impaired attention, difficulty in item naming, writing, agraphia, and executive dysfunction, aphasia, and confusion [38,39,41,43,44]. Montreal cognitive assessment (MoCA) is an easy and widely used test evaluating the following: (1) short-term memory; (2) visuospatial abilities; (3) higher cerebral functions, such as attention, concentration, and working memory; (4) language; (5) orientation to place and time [47,48]. MoCA is helpful for the early detection of cognitive dysfunction [49,50]. Mohn et al. used the MoCA test for cognitive evaluation during a 10-day period post-infusion in a cohort of 15 patients treated with tisagenlecleucel: in 73.4% (11/15) of the study participants, test values were inside the normal values during the whole study period, while the other four were diagnosed with ICANS [48]. Sales et al. showed in their study that the MoCA test is more reliable for the identification of patients with cognitive dysfunction compared to the ICE score [49]. In this study, 12 of the 53 patients developed neurotoxicity, while 10 of them received axicabtagene ciloleucel and two were treated with tisagenlecleucel. However, Herr et al. noticed in a series of patients that cognitive changes (personality alteration, occupational confusion, or inability to answer questions) were not associated with ICE score [45]. Thus, a three-step command tool for the early identification of neurotoxicity was developed to be used in conjunction with the ICE score [45].

Dimensional change card sort (DCCS) is a method for evaluation of the higher cerebral functions: participants are asked to classify a series of divalent cards, firstly according to one category (such as color), and then according to another (such as shape) [51,52]. A helpful for working memory assessment might be the list sorting working memory test (LSWMT). In this test, the participant is expected to remember and order different items that are presented both visually and via audio [53]. Moreover, the Flanker inhibitory control and attention test (FICAT) has been developed for the evaluation of attention and inhibitory control. The patient’s attention has to be focused on a certain stimulus while restraining attention to the stimuli flanking it [54]. The Processing Speed Index (PSI) is used for the speed of cognitive procedure and response output appraisal [51,55]. Shalabi et al. were among the first to study neurocognitive outcomes in children and young adults who receive CAR-T cell therapy using DCCS, FICAT, LSWMT, and Wechsler PSI tests and comparing mean scores between the baseline and 21–28 days post-treatment [51]. In this study, 22 patients were enrolled, 21 with acute lymphoblastic leukemia. DCCS, FICAT, and LSWMT mean scores did not significantly differ post-treatment in comparison to the baseline, while the Wechsler PSI score was found to be significantly improved (*p* = 0.048, t = 2.15).

The quality of life in neurological disorders questionnaire (NeuroQoLv2) is an easy, reliable, and patient-reported measurement system for the evaluation of the mental and physical health of individuals with neurological diseases, which has been administered as well to CAR-T cell patients [56,57]. Sidana et al., in their cohort of 34 patients, estimated the NeuroQoLv2 at baseline, 2 weeks after the infusion, and monthly for 6 months post-treatment [57]. They reported a significantly improved NeuroQoLv2 score at 4 months post-infusion in comparison to the baseline. Similarly, in an observational study, 163 patients’ cognition performance was evaluated at different time points (at baseline, 3 months, 12 months) with an everyday cognition questionnaire [58]. Cognition mean levels did not change from the baseline to 3 months post-treatment (*p* > 0.05). However, from 3 months to 12 months, patients reported a decline in mean levels of memory (*p* = 0.04, d = 0.15), global cognition (*p* = 0.01, d = 0.18), organization (*p* = 0.03, d = 0.17), language (*p* = 0.04, d = 0.15), and divided attention (*p* = 0.001, d = 0.28). MD Anderson Symptom Inventory (MDASI) is a patient-reported outcome measurement system for cancer-related symptoms, examining 13 items, among which are memory deficits [59]. Wang et al. administered the MDASI questionnaire to 60 CAR-T recipients and they did not identify a statistically significant difference in the cognition/difficulty remembering question ratings between different time points [60]. Furthermore, both the interviewing of patients with questions concerning cognition and the recording of patient-reported outcomes have also been used for this purpose in clinical practice [58,61,62,63]. Cheng et al. conducted a qualitative study investigating retrospective patient-reported cognitive symptoms: five, 10, and one participant(s) reported cognitive symptoms before CAR-T infusion, at baseline, and 6 months after the treatment, respectively [62]. In another qualitative study, 60% of the study participants were found to face cognitive problems and mainly memory deficits (2 to 6 months post-infusion) [63]. The European Organization for Research and Treatment of Cancer Quality-of-Life Q30 Questionnaire (QLQ-C30) has also been used for the measurement of neurocognitive outcomes in these patients [13,40,42,46]. Delforge et al., using QLQ-C30, showed that self-reported cognition improved 2–9 months after the infusion, and the improvement persisted even 18 months post-infusion [64].

The Digit Span (DS) test from the Wechsler adult intelligence scale has been also implemented for the working memory evaluation of these patients [65,66,67,68]. Furthermore, the free and cued selective reminding test (FCSRT) is useful for both the evaluation of verbal episodic memory and the detection of dementia [64,65,69,70]. The prospective and retrospective memory questionnaire (PRMQ) consists of 16 questions, eight examining prospective memory failures, and eight regarding retrospective failures [66,67,71]. The Trail Making Test (TMT), a neuropsychological test for the assessment of visual scanning and task switching, has been applied for the neurocognitive function evaluation of CAR-T cell therapy survivors [65,66]. TMT consists of two parts, in which the patient has to draw a line between 24 consecutive circles randomly allocated on the page, and can be completed in 5 to 10 min [72,73,74]. In the Rey–Osterrieth complex figure (ROCF) test the patient is asked to draw a complicated figure; thus, the functional decline in multiple cognitive dimensions, such as attention, visual memory, and concentration can be assessed [65,66,75]. The praxis test is a gesture-based diagnostic test for cortical pathologies such as Alzheimer’s disease, in which participants have to imitate certain gestures [65,66,76]. The Boston diagnostic aphasia examination (BDAE) has been developed for the evaluation of patients with suspected aphasia and related disorders [65,66]. BDAE administration lasts from 20 to 120 min and examines five items: (1) conversational and descriptive speech; (2) acoustic comprehension; (3) articulation; (4) reading; and (5) writing [77]. Moreover, Mini-mental state examination (MMSE) testing is an easy way to rapidly assess cognitive function in everyday clinical settings [78]. MMSE consists of 11 questions assessing five areas of cognitive function: orientation, registration, attention and calculation, language, and recall [79]. The highest score is 30, while a score equal to or below 23 is considered suggestive of cognitive impairment. MMSE testing has been implemented for cognition evaluation in these patients, mainly in a 6-month period post-infusion [47,65,66]. Assessment of the ability to name items from a given category within a fixed time, known as semantic fluency, has also been used for cognitive dysfunction detection in CAR-T cell therapy survivors [65,66]. Semantic disfluency is prevalent in patients with Parkinson’s disease and is indicative of a higher risk of dementia development [80]. Moreover, another method that has been utilized for this purpose is DO-80, an oral denomination test of 80 images [65,66]. However, DO-80 is only available in some languages and payment is required for its use [81]. Maillet et al., in their observational study, assessed cognitive functions (memory, executive functions, language, and praxis), in 56 patients at baseline (5 days before CAR-T infusion), 6-, and 12-months post-infusion, using the above-mentioned tests [65]. They did not find a significant decline in comparison to the baseline in the evaluated cognitive functions, while scores in tests for visuo-construction (*p* < 0.001), visuospatial ability (*p* < 0.001), and short-term memory (*p* = 0.002) were improved during follow-up. Moreover, in a cohort of 19 disease-free patients, no statistically significant differences were reported between baseline and follow-up (2 years post-infusion) in any of the 10 neuropsychological tests conducted [66].

The Continuous Performance Test 3rd Edition (CPT3) is a computerized evaluation for attention-related problems that has been used in these patients [82,83]. Another method described is the Stroop color and word test (SCWT) [65,66,82]. Repeatable battery for the assessment of neuropsychological status (RBANS) evaluating both cognitive function and neuropsychological status has also been administered in the patients for the evaluation of neurocognitive performance [82,84]. SCWT is a cognitive test examining the ability of the participant to inhibit cognitive interference which happens when the processing of a certain stimulus feature blocks the concurrent processing of a second stimulus attribute [85]. The Wechsler test of adult reading (WTAR) has been implemented to assess the pre-infusion intellectual function of the patients [82,86]. Hoogland et al., in a cohort of 117 patients, assessed total neurocognitive performance (TNP) and cognitive functions at baseline, 1-, 3-, and 12-months post-treatment, using, among others, WTAR, Stroop color, RBANS, and CPT3 [82]. TNP and executive function decreased slightly from baseline to 3 months post-infusion and improved at 12 months (*p* < 0.04), while slight but significant linear declines in visuospatial ability were also noticed (*p* = 0.03). In another study examining four self-reported cognitive outcomes, 37.5% of the patients reported one or more cognitive difficulties (35% memory, 30% word finding, 22.5% concentration, 12.5% problem solving) [61]. A total of 10% of study participants experienced all four cognitive difficulties after CAR-T cell therapy [61]. Furthermore, the Hopkins verbal learning test (HVLT) is used for the assessment of memory and verbal learning even in individuals with cognitive impairment [87]. A Cog-12 questionnaire is used for cognitive function assessment, and the neuropsychiatric inventory questionnaire (NPI-Q), which measures the presence and severity of 12 neuropsychiatric symptoms in individuals with dementia, has been applied for the neurocognitive evaluation of CAR-T recipients [47,88,89]. Wang et al. published a case report, in which the patient’s memory (especially for recent events) declined one month post-CAR T-cell therapy after the occurrence of a seizure [47]. These findings continued even two years post-treatment, as shown by neuropsychological assessment (MMSE, MoCA, HVLT, Cog-12, and NPI-Q tests) [47].

In Table 1, the results of studies examining the long-term cognitive outcomes of CAR-T therapy survivors are summarized. As shown, most of the cognition outcomes remain stable, while some patients state a decline in their memory, while still others state an improvement.

**Figure 2:**
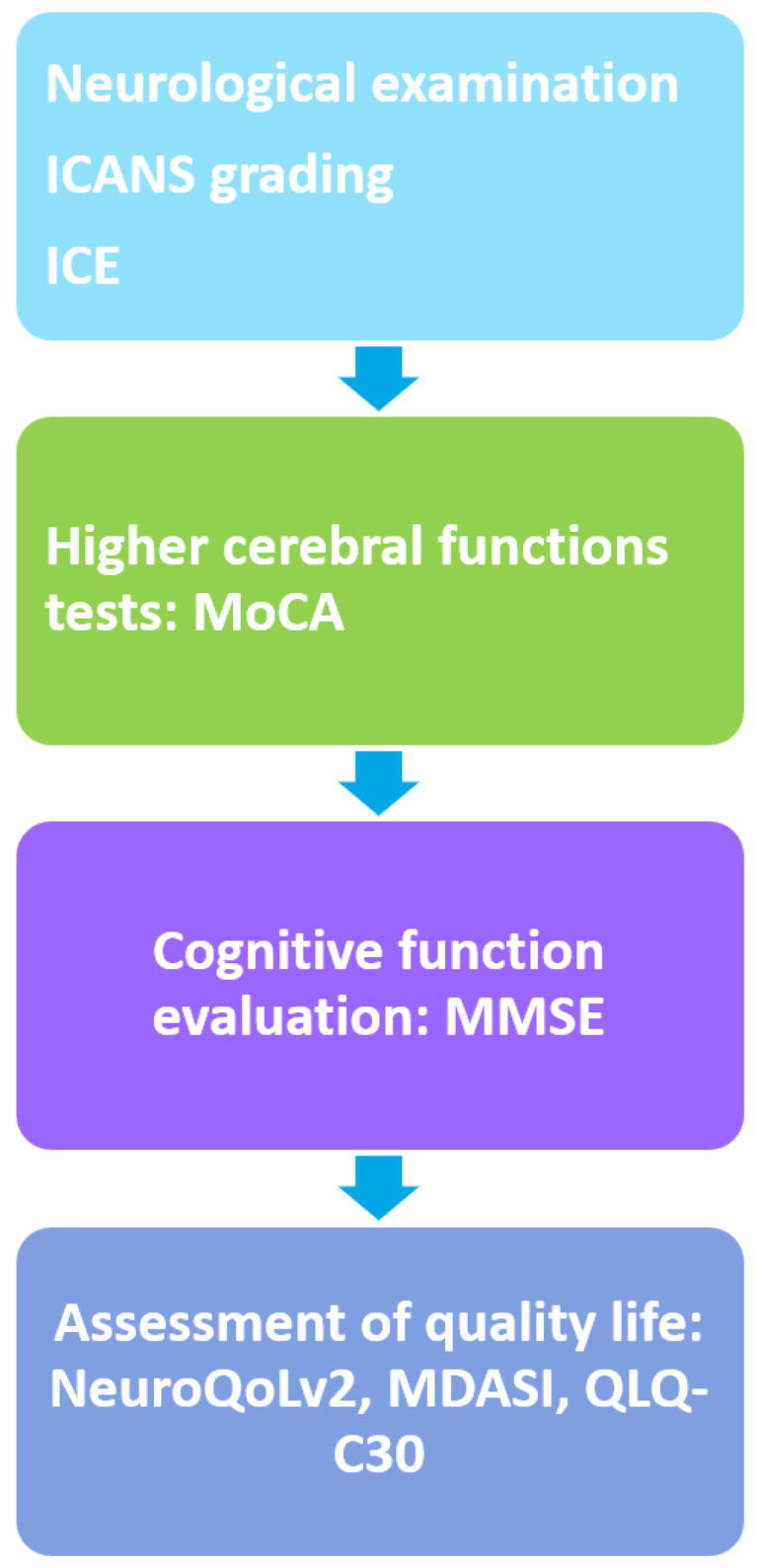
Diagnostic tests for rapid evaluation of neurocognitive outcomes in patients who receive CAR-T cell therapies in everyday clinical practice. CAR-T: chimeric antigen receptor T; MMSE: Mini-mental state examination; MoCA: Montreal cognitive assessment; NeuroQoLv2: quality of life in neurological disorders questionnaire; MDASI: MD Anderson Symptom Inventory; QLQ-C30: European Organization for Research and Treatment of Cancer Quality-of-Life Q30 Questionnaire.

Summarizing neurological clinical examination, ICANS grading with ICE score, semi-structured interviews with the patients, focusing on their self-reported cognitive issues, use of questionnaires, and other diagnostic tests are crucial for the early detection of neurocognitive impairment at different time points (pre- and post-infusion). This approach for application in everyday clinical practice is presented in Figure 2. ICANS grading is an easy way to assess neurocognitive function in the early period after CAR-T infusion. The MoCA test can also be used pre- and post-infusion to evaluate the differences in cognition and higher cerebral functions, because it is easy, rapid, and can be performed by non-specialized staff. Moreover, MMSE can be applied at bedside and might be used long-term, during follow-up, to detect cognitive impairment. Semantic fluency should also be tested. Questionnaires examining quality of life in CAR-T immunotherapy survivors are essential to improve the daily life of this population. Semi-structured interviews and qualitive studies are of paramount importance but might be time-consuming in everyday practice. The rest of the tests might be performed by experienced neurocognitive sciences staff, given their complexity. Thus, multidisciplinary teams are essential in this field.

## 4. Risk Factors and Therapeutic Targets for Cognitive Impairment following CAR-T Cell Therapy: The Unanswered Question

To date, no risk factors have been associated with the development of cognitive impairment post-CAR-T infusion. Patient age at infusion has not been correlated with neurocognition dysfunction onset both in pediatric and adult patients [44,51,58,82]. Hoogland et al. examined the length of hospitalization as a risk factor among others, but they failed to show an association with cognitive dysfunction onset [82]. Moreover, no association has been identified between cognitive outcomes and the number of previous therapy lines, sex, CRS, and ICANS grade [58,61,82]. Studies with a large number of participants and efforts for association with other risk factors, such as malignancy type, CAR-T product, previous allogeneic or autologous hematopoietic stem cell transplantation, and infectious complications are crucial to shed light in this field.

Further research is warranted for identifying potential preventative or therapeutic targets of cognitive deficits following CAR-T cell therapy. Wang et al., in their case report, presented a patient with cognitive impairment (memory loss) post-CAR-T infusion [47]. Oxiracetam (0.8 g orally twice daily) for 5 months was prescribed to the patient and an improvement in his memory was observed. However, after the discontinuation of oxiracetam, his cognition declined again. Administration of sodium oligomannate (GV-971) (450 mg orally twice daily) was subsequently decided, with a significant improvement in the overall condition of the patient and on MMSE and MoCA test scores. These data have to be confirmed in large patient cohorts both with randomized clinical trials and real-world data. Anakinra (interleukin 1-β receptor antagonist) and defibrotide (an agent used for endothelial injury) have been investigated as potential preventive agents for ICANS in phase I and II trials [90,91,92,93]. It would be interesting to examine whether the prophylactic use of these agents acts as a prevention for cognitive impairment in CAR-T cell recipients.

## 5. Conclusions and Future Directions

Cognitive deficits are a common complication that CAR-T cell recipients experience, especially during the first week post-treatment. During the follow-up, most of the cognition outcomes remain stable, while some patients report a decline in their memory while others an improvement. More studies, providing real-world evidence, are essential to make reliable conclusions.

The close liaison between neurologists, hematologists, nurses, and psychologists is crucial for the early detection of neurocognitive dysfunction in these patients. Monitoring of neurological symptoms, estimation of ICE score to grade ICANS, and use of standardized neurocognitive scores, such as MoCA and MMSE, can be helpful for the early recognition of cognitive impairment. Moreover, semi-structured interviews to collect self-reported neurocognitive outcomes from the patients are considered important to investigate the prevalence and manifestations of cognitive impairment post-CAR-T infusion. Long-term monitoring is also vital to improve patients’ quality-of-everyday-life development, and guidelines about long-term neurocognitive monitoring of CAR-T cell therapy survivors are needed.

Future research has to focus on the early recognition of cognitive dysfunction, the identification of risk factors associated with it, and a better understanding of the pathophysiology of this impairment. Cognitive impairment, and especially memory loss, has to be investigated to ascertain whether it is associated with endothelial damage and microvascular alterations, as has been suggested for other clinical entities [94,95,96]. In the era of precision medicine, the prevention of neurocognitive dysfunction is substantial to improve the outcomes of our patients, and novel technologies, such as artificial intelligence models might be helpful in this direction [97,98].

## Figures and Tables

**Table 1:** Studies examining long-term cognition (>6 months) outcomes in CAR-T cell therapy recipients.

First Author, Year of Publication	Type of Hematological Malignancy	Study Population (N)	Age of Study Participants	Tests for Cognition Evaluation	Outcomes
Ruark (2020) [61]	Relapsed/ refractory B-acute lymphoblastic leukemia, non-Hodgkin lymphoma, chronic lymphocytic leukemia	40	Median = 54 (range 22–74)	Self-reported questions on cognition	37.5% of the participants reported one or more cognitive difficulties (35% memory, 30% word finding, 22.5% concentration, 12.5% problem solving)
Maillet et al. (2021) [65]	Relapsed/ refractory diffuse large B cell lymphomas	56	Mean = 58 (standard deviation ±14)	MMSE, BDAE, DO-80, semantic fluency, digit span, TMT, Stroop test, FCSRT, ROCF, praxis scale, PRMQ	Scores in tests for visuo-construction (*p* < 0.001), visuospatial ability (*p* < 0.001), and short-term memory (*p* = 0.002) were improved during follow-up.
Ursu et al. (2022) [66]	Relapsed non-Hodgkin lymphomas	56	Median = 69 (range 26–72)	MMSE, semantic fluency, DO-80, BDAE, digit span, TMT, Stroop test, FCSRT, ROCF, praxis scale, PRMQ	No statistically significant difference was reported between baseline and follow-up (2 years post-infusion) in any of the neuropsychological tests conducted.
Hoogland et al. (2022) [82]	Relapsed non-Hodgkin lymphomas	117	Mean = 60.92 (standard deviation ±11.57)	Color trails, WTAR, RBANS, CPT3, Stroop test	TNP and executive function decreased slightly from baseline to 3 months post-infusion and improved at 12 months (*p* < 0.04), while slight but significant linear declines in visuospatial ability were also reported (*p* = 0.03).

CAR-T: chimeric antigen receptor T; MMSE: Mini-mental state examination; BDAE: Boston diagnostic aphasia examination; TMT: Trail Making Test; FCSRT: free and cued selective reminding test; ROCF: Rey–Osterrieth complex figure; PRMQ: prospective and retrospective memory questionnaire; RBANS: repeatable battery for the assessment of neuropsychological status; CPT3: Continuous Performance Test 3rd Edition.
